# Outbreak-Related Disease Burden Associated with Consumption of Unpasteurized Cow’s Milk and Cheese, United States, 2009–2014

**DOI:** 10.3201/eid2306.151603

**Published:** 2017-06

**Authors:** Solenne Costard, Luis Espejo, Huybert Groenendaal, Francisco J. Zagmutt

**Affiliations:** EpiX Analytics, Boulder, Colorado, USA (S. Costard, H. Groenendaal, F.J. Zagmutt);; Consultant, St. Augustine, Florida, USA (L. Espejo)

**Keywords:** milk, cheese, raw foods, pasteurization, risk assessment, risk, foodborne diseases, foodborne illnesses, disease outbreaks, food safety, public health, *Escherichia coli*, *E. coli*, *Salmonella*, *Listeria*, *Campylobacter*, bacteria, United States

## Abstract

The growing popularity of unpasteurized milk in the United States raises public health concerns. We estimated outbreak-related illnesses and hospitalizations caused by the consumption of cow’s milk and cheese contaminated with Shiga toxin–producing *Escherichia coli*, *Salmonella* spp., *Listeria monocytogenes*, and *Campylobacter* spp. using a model relying on publicly available outbreak data. In the United States, outbreaks associated with dairy consumption cause, on average, 760 illnesses/year and 22 hospitalizations/year, mostly from *Salmonella* spp. and *Campylobacter* spp. Unpasteurized milk, consumed by only 3.2% of the population, and cheese, consumed by only 1.6% of the population, caused 96% of illnesses caused by contaminated dairy products. Unpasteurized dairy products thus cause 840 (95% CrI 611–1,158) times more illnesses and 45 (95% CrI 34–59) times more hospitalizations than pasteurized products. As consumption of unpasteurized dairy products grows, illnesses will increase steadily; a doubling in the consumption of unpasteurized milk or cheese could increase outbreak-related illnesses by 96%.

Consumer demand for organic and natural foods (i.e., minimally processed foods) has been on the rise (*1*). However, in contrast to some perceptions (*2*), natural food products are not necessarily safer than conventional ones, as evidenced by higher rates of foodborne illnesses associated with unpasteurized dairy products (*3*–*6*). Pasteurization has greatly reduced the number of foodborne illnesses attributed to dairy products, and continuous efforts to reduce milk contamination pre- and post-pasteurization are further decreasing the disease burden (*3*). Yet, despite a decrease in dairy consumption in the United States (*7*), recent studies (*3*,*6*) suggest that over the past 15 years the number of outbreaks associated with unpasteurized dairy products has increased. In parallel with this increase, an easing of regulations has facilitated greater access of consumers to unpasteurized milk (e.g., through farm sales or cow share programs). The number of states where the sale of unpasteurized milk is prohibited decreased to 20 in 2011 from 29 in 2004 (*8*–*10*). This trend toward increased availability of unpasteurized dairy products raises public health concerns, especially because raw milk consumers include children (*2*,*4*,*6*).

Our study aimed at estimating the outbreak-related disease burden associated with the consumption of fluid cow’s milk and cheese made from cow’s milk (herein also referred to as milk and cheese or dairy products) that are unpasteurized and contaminated with *Campylobacter* spp., *Salmonella* spp., Shiga toxin–producing *Escherichia coli* (STEC), and *Listeria monocytogenes*. We also assessed how hypothetical increases in unpasteurized dairy consumption would affect this outbreak-related disease burden.

## Methods

### Data Sources

**Figure 1:**
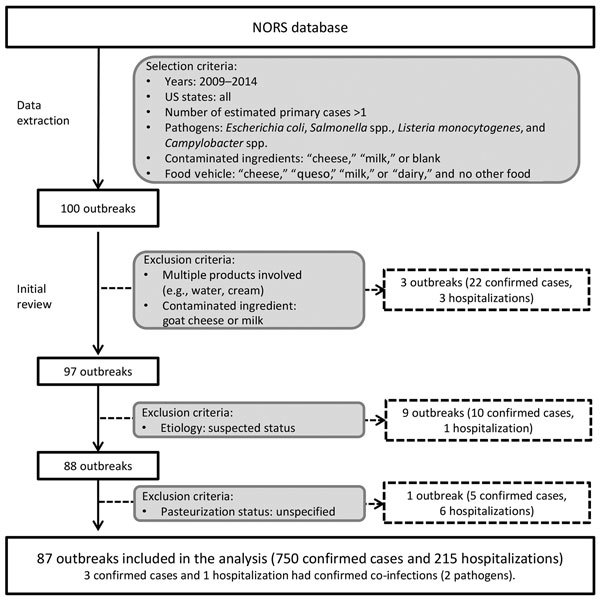
Process for selecting US outbreaks associated with cow’s milk and cheese, 2009–2014. Laboratory-confirmed cases are cases with illness in which a specimen was collected and a laboratory was able to confirm the pathogen(s) or agent(s) causing illness. Hospitalizations are cases in which the patient was hospitalized as a result of becoming ill during the outbreak. NORS, National Outbreak Reporting System.

We used outbreak data from the National Outbreak Reporting System (NORS) (*11*) to estimate the incidence rates of illnesses and hospitalizations. NORS is a web-based platform that stores data on all foodborne disease outbreaks reported by local, state, and territorial health departments in the United States that have occurred since 2009. We included all outbreaks that occurred during 2009–2014 in which the confirmed etiologic agents were any of the 4 pathogens of interest (*Campylobacter* spp., *Salmonella* spp., STEC, and *L. monocytogenes*) and the implicated food vehicle or contaminated ingredient was milk or cheese (Figure 1). Outbreaks associated with multiple products; processed dairy products other than milk and cheese (e.g., cream, butter, yogurt, and kefir); milk produced by species other than cows; and cheese originating from species other than cows were excluded from the analysis (Technical Appendix 1). In addition, outbreaks with a suspected etiology status or associated with a dairy product with an unknown pasteurization status were excluded. 

**Figure 2:**
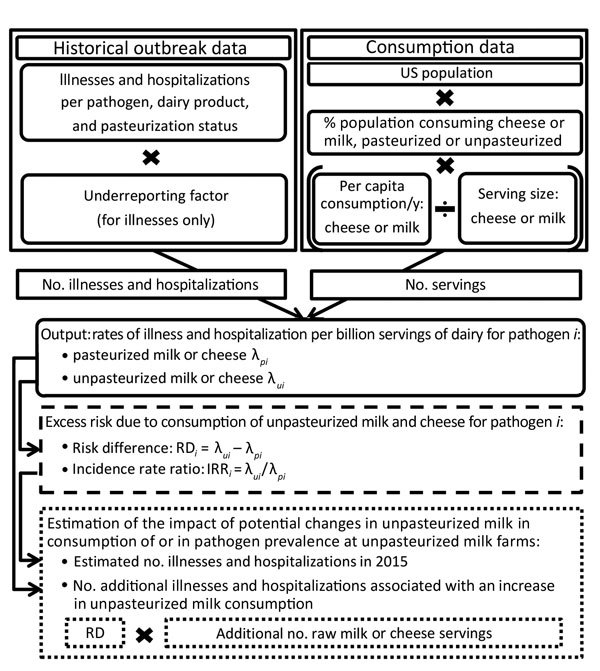
Stochastic model used to estimate the excess risk of outbreak-related illnesses and hospitalization due to unpasteurized dairy product consumption in the United States, 2009–2014. Model contains 3 main components: estimation of the incidence rates of illness and hospitalization for pasteurized and unpasteurized dairy products (elements in the boxes with solid lines), estimation of the excess risk associated with unpasteurized milk or cheese consumption (elements in box with dashed lines), and evaluation of the impact of hypothetical changes in consumption of unpasteurized dairy products (elements in boxes with dotted lines).

The stochastic model (Figure 2) was developed to estimate the following: the incidence rates of illness and hospitalization for pasteurized and unpasteurized dairy products, the excess risk associated with unpasteurized milk and cheese consumption, and the effect potential increases in consumption of unpasteurized dairy products would have on the outbreak-related disease burden (Technical Appendix 2 Tables 1–5). Inputs (other than the outbreak data) used in the stochastic model were derived from readily available sources of information (Technical Appendix 2). Dairy consumption estimates were derived from the Foodborne Active Surveillance Network (FoodNet) Population Survey (*12*).

### Estimation of the Incidence of Outbreak-Related Illnesses and Hospitalizations

We modeled the uncertainty of the pathogen-specific and pasteurization status–specific incidence rates of illness and hospitalization (λ) in the United States per serving of dairy product using a conjugate gamma distribution (*13*). The number of hospitalizations and laboratory-confirmed cases occurring during the study period (2009–2014) that were caused by a given pathogen after consumption of milk or cheese of a certain pasteurization status was obtained from the NORS database. For laboratory-confirmed cases, this number was adjusted for underreporting, under testing (only a proportion of suspected cases were sampled and tested), and underdiagnosis (based on diagnostic test sensitivity), in order to estimate illnesses for 2009–2014. These pathogen-specific factors were assumed to be independent of the product consumed and its pasteurization status, and constant for the years considered. The analysis did not include adjustment factors for potential misclassification in terms of etiology or pasteurization status. These 2 outbreak characteristics were carefully reviewed, and any outbreak for which the information could not be verified was excluded. It was thus assumed that etiology and pasteurization status misclassifications were negligible in this analysis.

Because NORS is a passive surveillance system, the inherent underreporting associated with it needed to be accounted for. We estimated an underreporting factor by using FoodNet data, which is an active surveillance system assumed to include virtually all identified cases (Technical Appendix 2). First, we extrapolated the total number of laboratory-confirmed cases in the US population during 2009–2013 using the incidence rates reported by FoodNet and considering the proportions of the US population included in FoodNet surveillance sites (*14*). Second, we estimated the total number of outbreak-related cases using the fraction of the US laboratory-confirmed cases that were outbreak-related (*15*). Third, we extracted the proportion of outbreak-related illnesses attributable to dairy (*16*). Fourth, we calculated the ratio of the number of outbreak-related, laboratory-confirmed cases linked to dairy consumption derived from the previously described calculations and the number of dairy-related, laboratory-confirmed cases reported through NORS to use as the underreporting factor in the analysis (Technical Appendix 2). When estimating the underreporting factor, we assumed that the FoodNet surveillance population and reporting practices were representative of the entire United States and that the food source attribution pertaining to the illnesses from confirmed and suspected outbreaks (*16*) were equally relevant to laboratory-confirmed cases from outbreaks of confirmed status only. We used the sensitivity of the diagnostic tests as described in Scallan et al. (*15*) to estimate the proportion of false-negative, laboratory-confirmed cases from NORS (underdiagnosis factor). Finally, we derived the under-testing factor by using the ratio of laboratory-confirmed primary cases to the estimated total number of primary illnesses reported to NORS (*17*).

The annual number of servings of milk or cheese of a given pasteurization status was calculated as the product of the number of servings of milk or cheese per person for a certain year, the resident population in the United States for that year (*18*) and the percentage of the population of dairy consumers that consume milk or cheese of a particular pasteurization status. The annual per capita consumption of a given dairy product (*19*) was divided by its average serving size (i.e., the amount of milk or cheese that is generally served) (*7*,*20*,*21*) to estimate the annual per capita number of servings of milk and cheese. These totals were then summed across the years of the study period. The per capita consumption data (*19*) were assumed to include both pasteurized and unpasteurized dairy products. Because unpasteurized dairy products constitute a small percentage of the total consumption, this assumption (if inaccurate) would likely have only a small effect on results. We also hypothesized that the serving sizes (*7*,*20*,*21*) were the same for pasteurized and unpasteurized dairy products.

The estimates of the proportion of dairy consumers that consume milk or cheese of a given pasteurization status were derived from the FoodNet Atlas of Exposure (*12*). Answers from this FoodNet survey are provided as aggregates per survey site, rather than per respondent. Therefore, answers regarding milk and cheese consumption were treated as independent. In addition, we assumed that respondents who reported consumption of unpasteurized milk or cheese did not consume pasteurized milk or cheese. Because the information to calculate the overall proportion of the US population consuming any type of cheese was unavailable, we assumed it to be equal to the proportion of the population reporting consumption of any cheese sold as or cut from solid blocks (i.e., the type of cheese consumed most commonly). We further assumed the proportion of the US population consuming unpasteurized cheese to be equal to the proportion reporting exposure to any cheese made from unpasteurized milk in the previous 7 days.

### Estimation of the Excess Risks Attributed to the Consumption of Unpasteurized Milk and Cheese

We estimated the additional risks for illness and hospitalization for consumers of unpasteurized dairy products compared with consumers of pasteurized ones. We calculated excess risk using 1) risk difference (RD), which measures the absolute difference in the observed risks for illness and hospitalization between consumers of unpasteurized dairy products and consumers of pasteurized ones, and 2) incidence rate ratio (IRR), which provides a relative comparison of the risks for illness and hospitalization between the 2 exposure groups (*22*).

### Effects of Hypothetical Changes in Consumption of Unpasteurized Milk or Cheese

We assessed the potential public health effects of hypothetical changes in unpasteurized milk consumption. We determined the number of illnesses in 2015 in the United States using the pathogen-specific rates of illnesses and hospitalizations per serving of dairy product. The number of hospitalizations was calculated as pathogen-specific fractions of these illnesses. The pathogen-specific probabilities of hospitalization in cases of illness were assumed unconditional on the pasteurization status of the dairy product involved, but rather dependent on the severity of illness (*23*,*24*).

We estimated the additional illnesses and the additional hospitalizations for each pathogen if a hypothetical increase in consumption of unpasteurized milk or cheese occurred using 1) the change in the proportion of the population consuming unpasteurized milk or cheese, 2) the number of servings of milk or cheese for 2015, and 3) the risk difference in illnesses per serving of dairy for that pathogen. We assumed that the overall proportion of the US population consuming milk or cheese did not change; therefore, the increase in the proportion of the US population consuming unpasteurized milk or cheese corresponded to a shift of dairy consumers from pasteurized to unpasteurized. Six hypothetical scenarios were considered: 10%, 20%, 50%, 100%, 200%, and 500% increases in the proportion of the US population consuming unpasteurized milk or cheese.

### Scenario and Sensitivity Analyses

We performed a sensitivity analysis to identify the parameters that most influenced our estimates. The sensitivity of the estimates to the input parameter uncertainties was calculated by using conditional means as implemented in @RISK 6.1.2 (Palisade Corporation, Ithaca, NY, USA). In addition, we assessed the robustness of our sensitivity analysis with a scenario analysis in which we calculated our estimates with different sets of outbreak data. For the main analysis, the model was run on outbreaks of confirmed etiology and pasteurization status. In the scenario analysis, the model was then re-run with either of the 2 following sets of outbreaks added to the main data set: outbreaks of suspected etiology status (*17*) and outbreaks involving dairy products of unspecified pasteurization status assumed to be caused by pasteurized dairy products.

### Model Implementation

The model was developed in Excel 2010 (Microsoft Corporation, Redmond, WA, USA) with the Monte-Carlo simulation add-in @RISK 6.1.2. Results are expressed as means and 95% credibility intervals (CrIs, a Bayesian equivalent to the confidence interval) or prediction intervals (PIs, which provides uncertainty bounds for predictions), unless stated otherwise.

## Results

### Incidence Rates and Increased Risks Associated with the Consumption of Unpasteurized Milk and Cheese

We used a total of 87 outbreaks causing 750 laboratory-confirmed illnesses and 215 hospitalizations in this analysis (Table 1). The incidence rates of STEC, *Salmonella* spp., and *Campylobacter* spp. illnesses and hospitalizations per 1 billion servings were higher for unpasteurized dairy product consumers than for pasteurized dairy product consumers. Illnesses and hospitalizations caused by *L. monocytogenes* infections were more often attributed to the consumption of pasteurized cheese than unpasteurized cheese (Table 2). Assuming no change in the consumption of unpasteurized dairy, dairy products contaminated with STEC, *Salmonella* spp., *L. monocytogenes*, and *Campylobacter* spp. were predicted to cause 761 (95% PI 598–994) outbreak-related illnesses and 22 (PI 13–32) hospitalizations in 2015. Unpasteurized dairy products caused 96% (PI 94%–98%) of these illnesses.

**Table 1:** Dairy-related illnesses and hospitalizations from 87 outbreaks, National Outbreak Reporting System, United States, 2009–2014*

Pathogen	Outbreaks associated with milk and cheese consumption, N = 87†						
	Pasteurized				Unpasteurized		
	Outbreaks	Illnesses	Hospitalizations		Outbreaks	Illnesses	Hospitalizations
STEC	0	0	0		14‡	99	42
*Salmonella* spp.	0	0	0		8§	83	29
*Listeria monocytogenes*	10	100	87		1	1	1
*Campylobacter* spp.	1	2	0		53‡§	465	56
Overall	11	102	87		76	648	128

*Illnesses and hospitalizations had confirmed etiologies and were associated with the consumption of milk or cheese of known pasteurization status. STEC, Shiga toxin–producing *Escherichia coli*. †Out of the 87 outbreaks, 10 outbreaks reported a total of 17 deaths, 16 of them were linked to *L. monocytogenes* and 1 to *Campylobacter* spp. ‡One outbreak (38 illnesses and 10 hospitalizations) had 3 cases with confirmed coinfection (STEC and *Campylobacter* spp.). These 3 cases were duplicated because they were assigned to each pathogen. §One outbreak (4 illnesses and 1 hospitalization) involved 2 pathogens: 3 Illnesses and 1 hospitalization were linked to *Campylobacter* spp. and 1 illness and 0 hospitalizations were linked to *Salmonella* spp.

**Table 2:** Incidence rates and risk differences for illness and hospitalization per 1 billion servings of milk or cheese, by pasteurization status and pathogen, United States, 2009–2014*

Pathogen	Illnesses				Hospitalizations		
	Unpasteurized	Pasteurized	Risk difference†		Unpasteurized	Pasteurized	Risk difference†
STEC	3.5 (2.7–4.5)	3.4 x 10^−4^ (3.1 x 10^−7^ to 1.7 x 10^−3^)	3.5 (2.7 to 4.5)		0.9 (0.6 to 1.2)	3.4 x 10^−4^ (3.0 x 10^−7^ to 1.7 x 10^−3^)	0.9 (0.6 to 1.2)
*Salmonella* spp.	49.1 (32.7–76.7)	3.4 x 10^−4^ (3.3 x 10^−7^ to 1.7 x 10^−3^)	49.1 (32.7 to 76.7)		0.6 (0.4 to 0.9)	3.5 x 10^−4^ (3.4 x 10^−7^ to 1.7 x 10^−3^)	0.6 (0.4 to 0.9)
*Listeria monocytogenes*	0.04 (0.003–0.100)	0.1 (0.08 to 0.12)	−0.06 (−0.11 to 0.02)		0.03 (2.2 x 10^−3^ to 0.1)	0.06 (0.05 to 0.07)	−0.03 (−0.06 to 0.04)
*Campylobacter* spp.	39.0 (30.8–48.3)	5.8x10^−3^ (2.4 x 10^−3^ to 1.1 x 10^−2^)	39.0 (30.8 to 48.3)		1.2 (0.9 to 1.5)	3.5 x 10^−4^ (3.5 x 10^−7^ to 1.7 x 10^−3^)	1.2 (0.9 to 1.5)
Overall	91.7 (71.8–120.9)	0.11 (0.09 to 0.13)	91.6 (71.7 to 120.8)		2.7 (2.2 to 3.3)	6.1 x 10^−2^ (4.9 x 10^−2^ to 7.5 x 10^−2^)	2.7 (2.2 to 3.2)

*Values are shown as mean incidence (95% credibility interval). STEC, Shiga toxin–producing *Escherichia coli*. †Excess risk is attributable to unpasteurized dairy.

**Figure 3:**
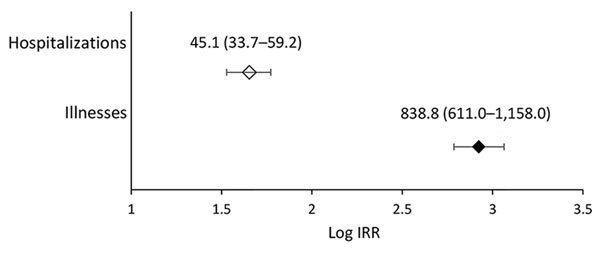
Forest plot showing, on a logarithmic scale, the excess risk for outbreak-related illnesses and hospitalizations caused by consumption of pasteurized and unpasteurized milk and cheese, United States, 2009–2014. Markers indicate mean log IRR of outbreak-related illnesses and hospitalizations caused by the food pathogens *Campylobacter* spp., *Listeria monocytogenes*, *Salmonella* spp., and Shiga toxin–producing *Escherichia coli* per 1 billion servings of unpasteurized milk or cheese relative to pasteurized products. Error bars indicate 95% credibility interval (CrI). Numbers above markers and bars are the IRR (not in log scale) and 95% CrI. log (IRR) = 0 indicates no difference in incidence rates between unpasteurized and pasteurized milk and cheese. IRR, incidence rate ratio.

We calculated the excess risk attributable to the consumption of unpasteurized milk and cheese (Table 2; Figure 3). Because no reported illnesses were caused by *Salmonella* spp. and STEC during 2009–2014 and no hospitalizations were caused by *Campylobacter* spp., the corresponding incidence rates were extremely low (Table 2). Therefore, only RDs (and not IRRs) were reported for these pathogens. If all milk and cheese consumed were pasteurized, an average of 732 (95% PI 570–966) illnesses and 21 (95% PI 12–32) hospitalizations would be prevented per year in the United States. Of these prevented cases, 54% would be salmonellosis and 43% campylobacteriosis. The mean IRR of illnesses was 838.8 (95% CrI 611.0–1,158.0) overall from all 4 pathogens of interest (Figure 3), with 0.4 (95% CrI 0–1.2) from *L. monocytogenes* and 7,601 (95% CrI 3,711–15,346) from *Campylobacter* spp. The rate of hospitalization was higher for unpasteurized dairy consumers than for pasteurized dairy consumers (mean IRR 45.1, 95% CrI 33.7–59.2), with an IRR of 0.5 (95% CrI 0–1.7) for *L. monocytogenes*.

### Effects of Hypothetical Scenarios

**Figure 4:**
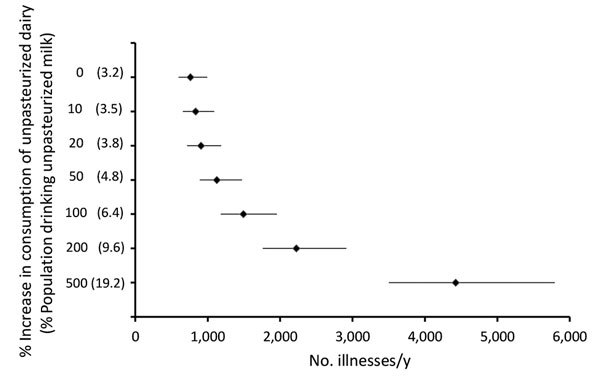
Number of dairy-related outbreak illnesses predicted per year in the United States if unpasteurized cow’s milk and cheese consumption increases 0%, 10%, 20%, 50%, 100%, 200%, and 500%. Numbers in parentheses indicate percentage of total population consuming unpasteurized cow’s milk. The illnesses graphed are those from outbreaks associated with cow’s milk or cheese contaminated with Shiga toxin–producing *Escherichia coli*, *Salmonella* spp., *Listeria monocytogenes*, and *Campylobacter* spp. Markers indicate means; bars indicate 95% prediction intervals. The consumption estimates were based on the year 2015, and a 0% increase corresponds to the current proportion of the US population consuming unpasteurized dairy products.

If the percentage of unpasteurized milk consumers in the United States were to increase to 3.8% and unpasteurized cheese consumers to 1.9% (i.e., an increase of 20%), the number of illnesses per year would increase by an average of 19% and the number of hospitalizations by 21%. If the percentages of unpasteurized milk and cheese consumers were to double, the number of illnesses would increase by an average of 96%, and the number of hospitalizations would increase by 104%, resulting in an additional 733 (95% PI 571–966) illnesses/year and 22 (95% PI 13–32) hospitalizations/year, which corresponds to a total of 1,493 (95% PI 1,180–1,955) illnesses/year (Figure 4), most caused by *Salmonella* spp. and *Campylobacter* spp.

### Scenario and Sensitivity Analyses

The following conditional means sensitivity analysis reports the change in the output mean if the input variable is set to its 5th and 95th percentiles while other inputs are sampled at random. The rates of illnesses (λ) caused by the consumption of unpasteurized milk and cheese were most sensitive to the underreporting factors (γ) for *Salmonella* spp. (mean range λ 34.9–72.5), *Campylobacter* spp. (mean range λ 33.1–45.3), and STEC (mean range λ 3.1–4.1), and at a secondary level to the undertesting (ρ) and underdiagnosis (μ) factors (results not shown). The overall IRR of illnesses was most sensitive to the underreporting factor for *Salmonella* spp. (mean range IRR 710.1–1,049.6). The number of illnesses per year caused by the consumption of milk or cheese was most sensitive to the rates of illnesses caused by *Salmonella* spp. and *Campylobacter* spp., as the main uncertainties apply to the incidence calculations for all pathogens (results not shown). Including the 9 outbreaks with a suspected-etiology status or the outbreak of unspecified pasteurization status (Figure 1) into the main analysis did not change the IRRs or the predicted number of illnesses or hospitalizations per year (results not shown). 

## Discussion

Unpasteurized dairy products are responsible for almost all of the 761 illnesses and 22 hospitalizations in the United States that occur annually because of dairy-related outbreaks caused by STEC, *Salmonella* spp., *L. monocytogenes*, and *Campylobacter* spp. More than 95% of these illnesses are salmonellosis and campylobacteriosis. Consumers of unpasteurized milk and cheese are a small proportion of the US population (3.2% and 1.6%, respectively), but compared with consumers of pasteurized dairy products, they are 838.8 times more likely to experience an illness and 45.1 times more likely to be hospitalized. Illnesses caused by *L. monocytogenes*, however, were found to be more often associated with the consumption of pasteurized cheese, albeit only causing 1 additional outbreak-related illness per year on average.

An easing of regulations has allowed greater access to unpasteurized milk in recent years (*8*–*10*), and this study shows that illnesses and hospitalizations will rise as consumption of unpasteurized dairy products increases. If such consumption were to double, the mean number of outbreak-related illnesses that occur every year would increase by 96%. Most unpasteurized dairy–related outbreaks are caused by pathogen contamination at the dairy farm (versus postpasteurization contamination for pasteurized products) (*3*); thus, one could assume that decreasing pathogen prevalence in bulk milk tanks on raw milk farms would help reduce illnesses. STEC has been found in 2.5% (95% CrI 0.1%–9.1%), *Salmonella* spp. in 4.6% (3.7%–5.6%), *L. monocytogenes* in 2.5% (0.1%–9.0%), and *Campylobacter* spp. in 4.7% (2.8%–7.0%) of bulk milk tanks on US raw milk farms (*25*–*29*). Given these low prevalences, strategies for further reduction are limited and involve multiple aspects of unpasteurized milk production (*30*). Boiling of milk before consumption seems to be a more realistic mitigation strategy, but this practice is unlikely to be implemented by unpasteurized dairy product advocates because it would affect the perceived benefits.

This study focused on the outbreak-related illnesses, which is only a fraction of all dairy-related illnesses in the United States. Two studies have documented the fraction of outbreak-related cases among FoodNet laboratory-confirmed cases (*15*,*31*); the fraction ranges from 0.5% for *Campylobacter* spp. to 19.0% for STEC according to Ebel et al. (*31*). These data suggest that the number of sporadic illnesses caused by contaminated dairy products in the United States might be much larger than that for outbreak-related illnesses. However, because of the lack of information on the characteristics of sporadic illnesses (such as food source attribution), we restricted the scope of this analysis to outbreak-related disease burden.

Our analysis relied on outbreak data from NORS (*11*), which is a passive reporting system affected by underreporting. We used dairy-related outbreak cases from FoodNet (*14*–*16*) as a comparison to estimate underreporting; therefore, any potential bias of this comparison was carried over to our estimation of outbreak-related illnesses. By extrapolating incidence rates of cases from the FoodNet catchment areas to the overall United States, we assumed that the FoodNet surveillance population and reporting practices were representative of the entire United States. However, the FoodNet catchment population represents only 15% of the US population from 10 nonrandom sites. Also, a recent study (*31*) suggested state-to-state variations in reporting practices; these variations might be even greater between FoodNet and non-FoodNet states. This difference might influence state-specific incidence rates or underreporting ratios, as well as other characteristics of the reported cases. For example, if a state reported the incriminated food source as the food item (e.g., homemade yogurt), it would not have been selected for inclusion in this analysis, but if they reported the ingredient used for preparation (e.g., in the case of homemade yogurt, fluid milk), it would have been included in our analysis. However, the size and direction of such biases and uncertainties associated with these complex surveillance systems (NORS and FoodNet) are difficult to quantify because of the paucity of data.

The rates of illnesses were most sensitive to the estimated underreporting factors, which were assumed to be associated with the severity of symptoms (*23*,*24*) and other factors, such as state health department resources, and thus independent of the pasteurization status. Also, because this analysis only considered outbreaks involving milk and cheese (and no other dairy products), we are probably underestimating the incidence of illnesses and hospitalizations. However, milk and cheese are the most commonly consumed dairy sources and cause the most outbreaks (milk and cheese caused 99% of dairy-related outbreaks reported to NORS during the study period), so the underestimation is likely limited. Nonetheless, the overall comparison of risk between consumers of pasteurized and unpasteurized products should remain valid.

Estimates of the proportion of the population consuming dairy products were derived from the FoodNet population survey (*12*). We assumed that respondents who reported consumption of unpasteurized milk or cheese were not consuming pasteurized dairy. However, if unpasteurized milk or cheese only represented a fraction of their dairy consumption, the number of servings of unpasteurized dairy products could have been overestimated, and thus the risk for consumers of unpasteurized dairy products might have been underestimated. Also, the FoodNet population survey is based on a relatively small convenience sample and might therefore not be accurate. For example, the self-reported estimates of consumption of unpasteurized milk and cheese (3.2% and 1.6%, respectively) (*12*) might be underestimates or overestimates, potentially caused by consumers confusing the terms raw, organic, and natural (or other reasons). In addition, consumption might have changed since the 2007 FoodNet population survey (*12*), which might have resulted in an under- or overestimation of the risk from unpasteurized milk products. However, because the proportion of dairy consumers using unpasteurized products remains small, and the IRRs are very large, this overestimation is likely limited, and the trend for additional illnesses as unpasteurized dairy consumption grows remains valid. Similarly, estimates of the consumption of pasteurized cheese are underestimates: data available only provide estimates of the highest exposure to a single type of cheese, rather than to any type of cheese (*12*), potentially resulting in a risk overestimation for consumers of pasteurized dairy products. This is a limitation, notably for outbreaks linked to queso fresco and other Mexican-style soft cheeses. Despite these limitations, to the authors’ knowledge, this study is based on the best available data and builds upon other well accepted risk attribution methods (*15*,*16*,*32*).

In conclusion, outbreaks linked to the consumption of cow’s milk and cheese were estimated to cause on average 761 illnesses and 22 hospitalizations per year in the United States. Unpasteurized products are consumed by a small percentage of the US dairy consumers but cause 95% of illnesses; the risk for illness was found to be >800 times higher for consumers of unpasteurized milk or cheese than for consumers of pasteurized dairy products. Therefore, outbreak-related illnesses will increase steadily as unpasteurized dairy consumption grows, likely driven largely by salmonellosis and campylobacteriosis.

Technical Appendix 1 Description of the stochastic model that provides equations and source information for the values used in the model. Outbreak-related illnesses and hospitalizations, consumption data, and population estimates aggregated over the study period (2009–2014).

Technical Appendix 2 Outbreak data from the National Outbreak Reporting System (NORS) used for the stochastic model. Outbreaks from 2009–2014, with *Campylobacter* spp., *Salmonella* spp., Shiga toxin–producing *Escherichia coli*, and *Listeria monocytogenes* as confirmed etiologic agents and with milk or cheese as the implicated food vehicle or contaminated ingredient.
