# Supplementary material for: Outbreak-Related Disease Burden Associated with Consumption of Unpasteurized Cow’s Milk and Cheese, United States, 2009–2014
Source: Emerg Infect Dis. 2017 Jun;23(6):957–64. doi: 10.3201/eid2306.151603 (PMC5443421; doi:10.3201/eid2306.151603)
Supplement: Technical Appendix 2 — Outbreak data from the National Outbreak Reporting System (NORS) used for the stochastic model. Outbreaks from 2009–2014, with Campylobacter spp., Salmonella spp., Shiga toxin–producing Escherichia coli, and Listeria monocytogenes as confirmed etiologic agents and with milk or cheese as the implicated food vehicle or contaminated ingredient. [file 15-1603-Techapp-s2.pdf]

Article DOI: https://dx.doi.org/10.3201/eid2306.151603

Outbreak-Related Disease Burden Associated with Consumption of Unpasteurized Cow's Milk and Cheese, United States, 2009–2014

Technical Appendix

Model structure

A stochastic model with 3 components was developed to estimate: the incidence rates of illness and hospitalization for pasteurized and unpasteurized dairy products, the excess risk associated with unpasteurized milk and cheese consumption, and the effect potential increases in consumption of unpasteurized dairy products would have on the outbreak-related disease burden. Estimations were stratified by pathogen and pasteurization status. For all equations below, Gamma distributions were parameterized as Gamma(Shape, Rate), and Beta distributions were parameterized as Beta(Shape 1, Shape 2). The parameterization of the Beta distributions used in this model assumes a noninformative Beta(1,1) prior to represent the lack of knowledge on the true value of p (i.e., p is equally likely to take values between 0 and 1).

Estimation of the Incidence Rate of Outbreak-Related Illness and Hospitalization

For each pathogen and dairy product of a given pasteurization status, the incidence rates of illness and hospitalization in the United States per serving of dairy product are estimated using a Bayesian conjugate of the Poisson rate parameter λ based on a noninformative prior $\lambda^{-0.5}$(*1*), approximated as Gamma(0.5, 0.00001), as follows:

$$\lambda \sim \text{Gamma}(\alpha + 0.5, N_{serving} + 0.00001) \text{ (equation 1)},$$

where α is the estimated number of outbreak-related illnesses or hospitalizations caused by the pathogen during 2009–2014, and $N_{serving}$ is the number of servings of milk or cheese.

For α, the number of hospitalizations were directly obtained from the National Outbreak Reporting System (NORS) (*2*), while the number of illnesses was obtained after correction for pathogen-specific underreporting, under testing (i.e., the fact that samples are not collected from all suspected cases and not all samples are tested), and underdiagnosis (i.e., false negative). Sets of independent adjustment factors were sampled and combined as shown below to estimate illnesses:

$$\alpha = \alpha_{obs} \times \gamma \times \mu \times \rho \text{ (equation 2),}$$

where α_{obs} is the number of laboratory-confirmed cases as reported in NORS (*2*), γ is the underreporting factor, μ is the underdiagnosis factor, and ρ is the under-testing factor for a given pathogen. Another model structure was tested, where the adjusting factors were modeled using a hypergeometric process. However, a sensitivity analysis showed this did not affect the results, and thus the more parsimonious model structure shown in equation 2 was chosen. Means and credibility intervals for the adjustment factors and the data used for their calculation are shown in online Technical Appendix Table 2.

Estimation of the Underreporting Factor γ

We estimated the underreporting factor by comparing the total number of laboratory confirmed cases from dairy-related outbreaks ($N_{ODRcases}$) reported to NORS from 2009 through 2013 in the United States with the estimated number of laboratory-confirmed cases from outbreaks that were attributed to dairy consumption from FoodNet ($N_{LCcases}$) for the same period:

$$\gamma = \frac{N_{LCcases}}{N_{ODRcases}} \text{ (equation 3).}$$

In doing so, we assumed that FoodNet surveillance population and reporting practices were representative of the overall United States. $N_{ODRcases}$ was directly obtained from NORS. $N_{LCcases}$ was derived from estimated numbers of laboratory-confirmed cases for the US population ($N_{UScases}$), and adjusted to outbreak and dairy-related cases:

$$N_{LCcases} = N_{UScases} \times P_{ORcases} \times P_{DRcases} \text{ (equation 4).}$$

$N_{UScases}$ was estimated by extrapolating the yearly incidence rates of laboratory-confirmed cases in the FoodNet population ($R_{UScases}$) to the US population N_{resUS} and summing them for 2009–2013:

$$N_{UScases} = R_{UScases} \times N_{resUS} \text{ (equation 5),}$$

where N_{resUS} was calculated from the FoodNet study population ($N_{FoodNet}$) and the proportion of the US population this study population represents ($P_{FoodNet}$):

$$N_{resUS} = \frac{N_{FoodNet}}{P_{FoodNet}} \text{ (equation 6).}$$

For the 4 pathogens of interest, the incidence rates of laboratory-confirmed cases in the FoodNet population ($R_{UScases}$) were given by:

$$R_{UScases} \sim \text{Gamma } (N_{FoodNetcases},\ N_{FoodNet}) \text{ (equation 7),}$$

where $N_{FoodNetcases}$ were the total number of laboratory confirmed cases reported by FoodNet. This estimated number of laboratory-confirmed cases in the US derived from FoodNet data ($N_{UScases}$) was then adjusted as described in equation 4, so as to only include the outbreak-related cases attributable to dairy.

Assuming that proportions of laboratory-confirmed cases that are outbreak-related ($P_{ORcases}$) are pathogen-specific and do not change over time, $P_{ORcases}$ were approximated using data from Scallan et al. (*3*):

$$P_{ORcases} \sim \text{Beta } (N_{ob} + 1\ ,\ N_{cases} - N_{ob} + 1) \text{ (equation 8),}$$

where N_{cases} was the total number of laboratory-confirmed cases, and N_{ob} was the number of these cases that were outbreak related, as reported to FoodNet for 2004–2008.

The pathogen-specific estimates of the proportion of outbreak-related illnesses that are attributable to dairy ($P_{DRcases}$) were derived from the study by Painter et al. (*4*):

$$P_{DRcases} \sim \text{Pert (minimum, most likely, maximun) (equation 9).}$$

This assumes that the proportion of outbreak-related illnesses caused by dairy products remained unchanged during 2004–2008 and 2009–2014 and that they applied to outbreaks associated with cow's milk and cheese only. The study by Painter et al. included complex and simple foods, but in the case of dairy products the large majority of outbreaks (99%) were caused by milk or cheese (i.e., simple foods) during our study period.

Estimation of the Underdiagnosis Factor μ

The underdiagnosis factor used in equation 2, μ, accounts for the rate of false negatives using the test sensitivity described in Scallan et al. (*3*):

$$\mu = 1 + (1 - Se) \text{ (equation 10),}$$

where

$$Se \sim \text{Pert (minimum, mode, maximum) (equation 11).}$$

Estimation of the Under-testing Factor ρ

The under-testing factor in equation 2, ρ, accounts for the fact that in an outbreak investigation, samples are not collected from all suspected cases, and diagnostic tests are not conducted on all samples taken:

$$\rho \sim 1/\text{Beta}\,(\alpha_{obs} + 1,\ \beta_{obs} - \alpha_{obs} + 1) \text{ (equation 12),}$$

where β_{obs} is the number of estimated primary cases, and α_{obs} is the number of laboratory-confirmed cases (*2*,*5*). Because of the clustering of the cases by outbreak, the above estimation could potentially be biased.

In equation 1, the number of servings of a given dairy product and pasteurization status, $N_{serving}$, was calculated as:

$$N_{serving} = N_{resid} \times N_{pers\ serv} \times p_{cons} \text{ (equation 13),}$$

where N_{resid} is the total resident population in the United States (online Technical Appendix Table 3), $N_{pers\ serv}$ is the number of servings per person, and p_{cons} is the proportion of the population of dairy consumers who consume milk or cheese of a given pasteurization status. For example, $p_{cons,milk,unpast}$, the proportion of the population of dairy consumers that consumes unpasteurized milk, is calculated as:

$$p_{cons_{milk,unpast}} = \frac{P_{UnPcons,milk}}{P_{UnPcons,milk} + P_{Pcons,milk}} \text{ (equation 14),}$$

with $P_{UnPcons,milk}$ being the proportion of the US population consuming unpasteurized milk and $P_{Pcons,milk}$ being the proportion of the US population consuming pasteurized milk. $N_{pers\ serv}$ is estimated from the per capita consumption, C_o (online Technical Appendix Table 4), and the mean serving size, s (online Technical Appendix Table 1):

$$N_{pers\ serv} = \frac{Co}{s}$$ (equation 15).

Estimation of the Proportion of the US Population Consuming Milk or Cheese of a Given Pasteurization Status, $P_{UnPcons}$ and P_{Pcons}

The estimates of the proportion of consumers of milk or cheese of a given pasteurization status in the United States was derived from the FoodNet Population Survey Atlas of Exposures 2006–2007 (*6*). P_{Pcons} was calculated as the weighted average of $P_{c,state}$, the FoodNet state-specific proportion of consumers of milk or cheese of a given pasteurization status, and w_{state}, the proportion of the FoodNet survey population that is from that given state (online Technical Appendix Table 5):

$$P_{Pcons} = \sum(P_{c,state} \times w_{state})$$ (equation 16).

$P_{c,state}$ is given by

$$P_{Pc,state} \sim \text{Beta}(N_{Pcons} + 1, N_{survey} - N_{Pcons} + 1)$$ (equation 17),

with N_{Pcons} being the number of respondents that indicated that they consumed the product in the last 7 days and N_{survey} the FoodNet survey population in the given state.

Estimation of the Excess Risks Associated with the Consumption of Unpasteurized Milk and Cheese

The additional risk of outbreak-related illness and hospitalization for consumers of unpasteurized dairy products, compared with consumers of pasteurized ones, was estimated using 2 measures of excess risk (*23*). The risk difference measures the actual difference in the incidence rates of illness and hospitalization between consumers of unpasteurized dairy products (λ_u) and consumers of pasteurized ones (λ_p):

$$\text{RD} = \lambda_u - \lambda_p$$ (equation 18).

The incidence rate ratio provides a relative comparison of the risks for illness and hospitalization between the 2 exposure groups:

$$\text{IRR} = \lambda_u / \lambda_p$$ (equation 19).

Impact of Hypothetical Changes in Consumption of Unpasteurized Milk or Cheese

A scenario analysis was performed for the year 2015 to assess the public health impact of hypothetical changes in consumption of unpasteurized dairy products. Six scenarios were considered: 10%, 20%, 50%, 100%, 200%, and 500% increases in the proportion of the US population consuming unpasteurized milk or cheese.

The number of annual outbreak-related illnesses associated with milk or cheese consumption, α_{pred}, was estimated as

$$\alpha_{pred} \sim \text{Poisson}(\lambda_u \times \mathrm{N}_{\text{serving,u}} + \lambda_p \times N_{serving,p}) \text{ (equation 20).}$$

As shown in equation 13, the number of servings of milk or cheese for 2015 requires the estimation of the total US resident population and the per capita consumption for that year. Using a simple linear regression, we predicted these 3 values using historical data on the US resident population from 1996 through 2014 (online Technical Appendix Table 3) and milk and cheese consumption per capita from 2006 through 2014 (online Technical Appendix Table 4). The variability in the 2015 predictions for these 3 values when considering parameter uncertainty was modeled using a standard prediction interval calculation:

$$y = b0 + \beta_t x_t + t(n-2)\, Sy\sqrt{1 + \frac{1}{n} + \frac{(x_t - \bar{x})^2}{SSx}} \text{ (equation 21),}$$

where y is the prediction for the year 2015, $b0$ is the regression intercept, β_t is the slope for the year (i.e., the yearly growth or decline in y), x_t is the predicted year (i.e., year 2015), $t(n - 2)$ is the Student's t distribution with a sample size n and $n - 2$ degrees of freedom. Sy is the standard deviation of the residuals, and SSx represents the sum of squares for x. Random samples from the previously described Student's t distribution were used to generate samples from equation 21.

Servings were then counted as pasteurized ($N_{serving,p}$) or unpasteurized ($N_{serving,u}$) depending on the relative proportions of the population of dairy consumers that are consuming products of a given pasteurization status. For example, for milk consumption we assumed that the proportion of the US population consuming unpasteurized milk ($P_{UnPcons,milk}$) increases by a certain percentage, P_{inc}, but the overall proportion of the US population consuming milk

(whether pasteurized or not) remains the same. Thus, we defined $\Delta P_{UnPcons}$, the change in the proportion of the population of dairy consumers that are eating unpasteurized milk, as

$$\Delta P_{UnPcons} = \frac{P_{inc} \times P_{UnPcons,milk}}{P_{UnPcons,milk} + P_{Pcons,milk}} \text{ (equation 22).}$$

And the fraction of milk servings that are unpasteurized milk servings is the sum of $P_{UnPcons,milk}$ and $\Delta P_{UnPcons}$.

The number of hospitalizations per year was modeled as a fraction of illnesses (α_{pred})

$$\alpha_{hosp} \sim \text{Binomial}(\alpha_{pred}, \rho_{hosp}) \text{ (equation 23),}$$

where the uncertainty in the probability of hospitalization in case of illness is modeled using the conjugate prior:

$$\rho_{hosp} \sim \text{Beta}(\alpha_{obshosp} + 1, \alpha - \alpha_{obshosp} + 1) \text{ (equation 24),}$$

where $\alpha_{obshosp}$ is the number of reported outbreak-related hospitalizations due to illnesses from a given pathogen.

Finally, the additional illnesses or hospitalizations following a hypothetical increase in consumption of unpasteurized milk or cheese were estimated as follows:

$$\alpha_{created} \sim \text{Poisson}[\text{RD} \times \Delta P_{UnPcons} \times \sum (N_{serving,p} + N_{serving,u})] \text{ (equation 25).}$$

References

1. United States Census Bureau. Population estimates. 2016 [cited 2016 Apr 29]. https://www.census.gov/programs-surveys/popest.html

2. Centers for Disease Control and Prevention. National Outbreak Reporting System (NORS). Atlanta (GA): Centers for Disease Control and Prevention; 2009. [cited 2016 Apr 29]. https://www.cdc.gov/nors/index.html

3. Scallan E, Hoekstra RM, Angulo FJ, Tauxe RV, Widdowson MA, Roy SL, et al. Foodborne illness acquired in the United States—major pathogens. Emerg Infect Dis. 2011;17:7–15. PubMed http://dx.doi.org/10.3201/eid1701.P11101

4. Painter JA, Hoekstra RM, Ayers T, Tauxe RV, Braden CR, Angulo FJ, et al. Attribution of foodborne illnesses, hospitalizations, and deaths to food commodities by using outbreak data, United States,

1998–2008. Emerg Infect Dis. 2013;19:407–15. PubMed http://dx.doi.org/10.3201/eid1903.111866

5. Centers for Disease Control and Prevention. NORS: National Outbreak Reporting System guidance. Atlanta (GA): Centers for Disease Control and Prevention; 2012. https://www.cdc.gov/nors/pdf/NORS_Guidance_5213-508c.pdf

6. Centers for Disease Control and Prevention. Foodborne Diseases Active Surveillance Network (FoodNet) population survey atlas of exposures, 2006-2007. Atlanta (GA): Department of Health and Human Services, Centers for Disease Control and Prevention [cited 2016 Sept 29]. https://www.cdc.gov/foodnet/surveys/FoodNetExposureAtlas0607_508.pdf

7. Centers for Disease Control and Prevention. Foodborne Diseases Active Surveillance Network (FoodNet). Atlanta (GA): Centers for Disease Control and Prevention. 2014 [cited 2016 Apr 29]. https://www.cdc.gov/foodnet/index.html

8. Stewart H, Dong D, Carlson A. Why are Americans consuming less fluid milk? A look at generational differences in intake frequency, ERR-149. 2013 May [cited 2015 Sep 29]. https://www.ers.usda.gov/webdocs/publications/err149/37651_err149.pdf?v=41423

9. Sebastian RS, Goldman JD, Enns CW, LaComb RP. Fluid milk consumption in the United States: what we eat in America, NHANES 2005–2006. 2010 Sep [cited 2016 Sept 29]. https://www.ars.usda.gov/ARSUserFiles/80400530/pdf/DBrief/3_milk_consumption_0506.pdf

10. Innovation Center for US Dairy. Diary’s role in the diet: Cheese. 2013 [cited 2017 Apr 05]. http://www.usdairy.com/DairyResearchInstitute/NHanes/Pages/Cheese.aspx

11. United States Department of Agriculture, Economic Research Service. Dairy data. 2014 [cited 2016 Apr 29]. http://www.ers.usda.gov/data-products/dairy-data.aspx#.UqJ_meJrfYh

12. Food Marketing Institute. Natural and organic foods. Arlington (VA): Food Marketing Institute; 2008. https://www.fda.gov/ohrms/dockets/dockets/06p0094/06p-0094-cp00001-05-Tab-04-Food-Marketing-Institute-vol1.pdf

13. Katafiasz AR, Bartlett P. Motivation for unpasteurized milk consumption in Michigan, 2011. Food Prot Trends. 2012;32:124–8.

14. Langer AJ, Ayers T, Grass J, Lynch M, Angulo FJ, Mahon BE. Nonpasteurized dairy products, disease outbreaks, and state laws–United States, 1993–2006. Emerg Infect Dis. 2012;18:385–91. PubMed http://dx.doi.org/10.3201/eid1803.111370

15. Robinson TJ, Scheftel JM, Smith KE. Raw milk consumption among patients with non–outbreak-related enteric infections, Minnesota, USA, 2001–2010. Emerg Infect Dis. 2014;20:38–44. PubMed http://dx.doi.org/10.3201/eid2001.120920

16. Claeys WL, Cardoen S, Daube G, De Block J, Dewettinck K, Dierick K, et al. Raw or heated cow milk consumption: review of risks and benefits. Food Control. 2013;31:251–62. http://dx.doi.org/10.1016/j.foodcont.2012.09.035

17. Mungai EA, Behravesh CB, Gould LH. Increased outbreaks associated with nonpasteurized milk, United States, 2007–2012. Emerg Infect Dis. 2015;21:119–22. PubMed http://dx.doi.org/10.3201/eid2101.140447

18. National Association of State Departments of Agriculture. Raw milk survey, November, 2004 [cited 2016 Apr 29]. http://www.nasda.org/File.aspx?id=1582

19. National Association of State Departments of Agriculture. NASDA releases raw milk survey. 2008 Apr 21 [cited 2016 Apr 29]. https://view.officeapps.live.com/op/view.aspx?src=http%3A%2F%2Fwww.nasda.org%2FFile.aspx%3Fid%3D2149

20. National Association of State Departments of Agriculture. NASDA releases raw milk survey. 2011 Jul 19 [cited 2016 Apr 29]. http://www.nasda.org/file.aspx?id=3916

21. Gelman A, Carlin JB, Stern HS, Dunson DB, Vehtari A, Rubin DB. Bayesian data analysis. 3rd ed. London: Chapman & Hall/CRC Press; 2013.

22. Gordis L. Epidemiology. 2nd ed. Philadelphia: W.B. Saunders; 2000.

23. Haas CN, Rose JB, Gerba CP. Quantitative microbial risk assessment. New York: John Wiley & Sons; 1999.

24. Interagency Microbiological Risk Assessment Guideline workgroup. Microbial risk assessment guideline: pathogenic organisms with focus on food and water. US Department of Agriculture, Food Safety and Inspection Service; Environmental Protection Agency; 2012. https://www.epa.gov/sites/production/files/2013-09/documents/mra-guideline-final.pdf

Technical Appendix Table 1. Model parameters, values, and references*

Parameter	Symbol	Parameter subgroup	Value		Source
No. US laboratory-confirmed cases from outbreaks related to milk or cheese consumption 2009–2013	$N_{ODRcases}$	Pathogen	No. confirmed cases		NORS database (*2*)
		Campylobacter spp.	*365*		
		Listeria monocytogenes	98		
		Salmonella spp.	72		
		STEC	92		
Population under surveillance (and corresponding % of the US population)	$N_{FoodNet}$ ($P_{FoodNet}$)	Year	No. under surveillance (% US population)		FoodNet (*7*)
		2009	46,859,541 (15.3)		
		2010	47,145,373 (15.2)		
		2011	47,505,580 (15.2)		
		2012	47,898,745 (15.3)		
		2013	48,231,023 (15.2)		
FoodNet cases 2009–2013	$N_{FoodNetcases}$	Year	No. *Campylobacter* spp. cases		FoodNet (*7*)
		2009	6,058		
		2010	6,372		
		2011	6,785		
		2012	6,812		
		2013	6,622		
		Year	No. *Listeria monocytogenes* cases		
		2009	157		
		2010	131		
		2011	141		
		2012	123		
		2013	123		
		Year	No. *Salmonella* spp. cases		
		2009	7,023		
		2010	8,273		
		2011	7,813		
		2012	7,842		
		2013	7,307		
		Year	No. STEC cases		
		2009	747		
		2010	896		
		2011	984		
		2012	1,090		
		2013	1,126		
Proportion of outbreak related cases	$P_{ORcases}$	Pathogen	Beta(Shape1; Shape2)	95% CrI	Scallan et al. (*3*)
		Campylobacter spp.	123; 28,757	0.4%–0.5%	
		Listeria monocytogenes	10; 643	0.7%–2.6%	
		Salmonella spp.	2122; 31,557	6.0%–6.6%	
		STEC	561; 2,934	14.9%–17.3%	
Proportion of dairy-related cases	$P_{DRcases}$	Pathogen	Pert(minimum; most likely; maximum)		Painter et al. (*4*)
		Campylobacter spp.	61.8; 64.8; 65.2		
		Listeria monocytogenes	15.7; 15.9; 16.3		
		Salmonella spp.	6; 7.2; 18.6		
		STEC	2.1; 2.3; 3		
Diagnostic test sensitivity	*Se*	Pathogen	Pert (minimum; mode; maximum)		Scallan et al. (*3*)
		Campylobacter spp.	0.6; 0.7; 0.9		
		Listeria monocytogenes	0.55; 0.71; 0.83		
		Salmonella spp.	0.6; 0.7; 0.9		
		STEC	0.6; 0.7; 0.9		
Under-testing factor 2009–2013	ρ	Pathogen	1/Beta(Shape1; Shape2)	95% CrI	NORS database (*2*,*5*)
		Campylobacter spp.	468; 435	1.82–2.06	
		Listeria monocytogenes	102; 16	1.09–1.25	
		Salmonella spp.	86; 10	1.06–1.21	
		STEC	100; 15	1.08–1.25	
Serving size of dairy product	*s*	Dairy product	Serving size, lb.		USDA-ERS surveys (*8–10*)
		Milk	4.86×10^{-1}		
		Cheese	7.44×10^{-2}		

*CrI, credibility interval; NORS, National Outbreak Reporting System; STEC, Shiga-toxin–producing *Escherichia coli*; USDA-ERS, United States Department of Agriculture Economic Research Service.

Technical Appendix Table 2. Adjustment factors (means and 95% CrI) used for the estimation of the incidence rates of outbreak-related illnesses

Pathogen	Underreporting (γ)	Underdiagnosis (μ)	Under-testing (ρ)
STEC	1.15 (1.00–1.35)	1.28 (1.17–1.38)	1.15 (1.08–1.25)
Salmonella spp.	19.58 (13.64–30.13)	1.28 (1.17–1.38)	1.12 (1.05–1.21)
Listeria monocytogenes	1*	1.30 (1.20–1.40)	1.16 (1.09–1.25)
Campylobacter spp.	1.61 (1.34–1.90)	1.28 (1.17–1.38)	1.93 (1.81–2.06)

*Our calculations comparing FoodNet and National Outbreak Reporting System data suggested that there was no underreporting of *L. monocytogenes*, probably because of the severity of cases. CrI, credibility interval; STEC, Shiga-toxin–producing *Escherichia coli*.

Technical Appendix Table 3. Total US resident population (N_{resid}), 1993–2014*

Year	Population, millions
1993	259.919
1994	263.126
1995	266.278
1996	269.394
1997	272.647
1998	275.854
1999	279.04
2000	282.193
2001	285.108
2002	287.985
2003	290.85
2004	292.805
2005	295.517
2006	298.38
2007	301.231
2008	304.094
2009	306.772
2010	309.33
2011	311.592
2012	313.914
2013	316.427
2014	318.907

* The total US population for most years are estimates from the US Census Bureau, with the exception of 2000 and 2010, which are results of the US census *(1)*.

Technical Appendix Table 4. Per capita consumption of milk and cheese (C_o), 2006–2014*

Year	Milk, lb.	Cheese, lb.†
2006	183.63	32.43
2007	181.20	32.94
2008	179.10	32.39
2009	178.46	32.48
2010	177.42	32.92
2011	173.86	32.23
2012	169.90	33.49
2013	165.03	33.63
2014	158.88	34.17

*Data from US Department of Agriculture Economic Research Service *(11)*.
†Total cheese (does not include ricotta cheese).

Technical Appendix Table 5. Probability density functions of the proportion of the population consuming pasteurized or unpasteurized milk and cheese ($P_{c,state}$) and percentage of FoodNet population (w_{state}) by state, 2006–2007*

	Proportion of population consuming milk				Proportion of population consuming cheese				
	Pasteurized		Unpasteurized		Pasteurized		Unpasteurized		
State	Beta(Shape1; Shape2)	95% CrI	Beta(Shape1; Shape2)	95% CrI	Beta(Shape1; Shape2)	95% CrI	Beta(Shape1; Shape2)	95% CrI	FoodNet population, %
CA	434; 132	73.1%–80.1%	34; 1,057	2.1%–4.2%	323; 204	57.1%–65.4%	28; 1,063	1.7%–3.6%	7.07
CO	723; 183	77.2%–82.4%	45; 1,798	1.8%–3.2%	624; 315	63.4%–69.4%	27; 1,816	1.0%–2.0%	5.88
CT	739; 178	78.0%–83.1%	50; 1,754	2.0%–3.6%	522; 367	55.4%–61.9%	30; 1,774	1.1%–2.3%	7.62
GA	720; 213	74.4%–79.8%	70; 1,743	3.0%–4.8%	489; 393	52.2%–58.8%	21; 1,792	0.7%–1.7%	20.77
MD	698; 233	72.1%–77.7%	56; 1,783	2.3%–3.9%	499; 411	51.6%–58.1%	27; 1,812	1.0%–2.0%	12.23
MN	785; 145	82.0%–86.7%	43; 1,773	1.7%–3.1%	549; 339	58.7%–65.0%	26; 1,790	1.0%–2.1%	11.31
NM	687; 219	73.0%–78.6%	61; 1,711	2.7%–4.4%	562; 306	61.6%–67.9%	45; 1,727	1.9%–3.3%	4.29
NY	744; 191	76.9%–82.1%	65; 1,775	2.8%–4.4%	541; 366	56.5%–62.8%	32; 1,808	1.2%–2.4%	9.29
OR	684; 216	73.2%–78.8%	51; 1,745	2.1%–3.7%	644; 254	68.8%–74.6%	26; 1,770	1.0%–2.1%	8.15
TN	723; 202	75.4%–80.7%	63; 1,715	2.7%–4.5%	456; 399	49.9%–56.6%	28; 1,750	1.0%–2.2%	13.4

*Data derived from the FoodNet Population Survey Atlas of Exposures (*6*). CrI, credibility interval.
